# Buffy Coat DNA Methylation Profile Is Representative of Methylation Patterns in White Blood Cell Types in Normal Pregnancy

**DOI:** 10.3389/fbioe.2021.782843

**Published:** 2022-01-05

**Authors:** Ranine Ghamrawi, Igor Velickovic, Ognjen Milicevic, Wendy M. White, Lillian Rosa Thistlethwaite, Julie M. Cunningham, Aleksandar Milosavljevic, Natasa M. Milic, Vesna D. Garovic

**Affiliations:** ^1^ Division of Nephrology and Hypertension, Department of Medicine, Mayo Clinic, Rochester, MN, United States; ^2^ Department of Medical Statistics and Informatics, Medical Faculty University of Belgrade, Belgrade, Serbia; ^3^ Department of Obstetrics and Gynecology, Mayo Clinic, Rochester, MN, United States; ^4^ Department of Perinatology, Olmsted Medical Center, Rochester, MN, United States; ^5^ Department of Molecular and Human Genetics, Baylor College of Medicine, Houston, TX, United States; ^6^ Department of Laboratory Medicine and Pathology, Mayo Clinic, Rochester, MN, United States

**Keywords:** buffy coat, DNA methylation, epigenetics, white blood cells, pregnancy

## Abstract

**Background:** We aimed to assess the extent to which the buffy coat DNA methylome is representative of methylation patterns in constitutive white blood cell (WBC) types in normal pregnancy.

**Methods:** A comparison of differential methylation of buffy coat DNA vs DNA isolated from polymorphonuclear (PMN) and lymphocytic fractions was performed for each blood sample obtained within 24 h prior to delivery from 29 normotensive pregnant women. Methylation profiles were obtained using an Illumina Human Methylation 450 BeadChip and CHaMP bioinformatics pipeline. A subset of differentially methylated probes (DMPs) showing discordant methylation were further investigated using statistical modeling and enrichment analysis.

**Results:** The smallest number of DMPs was found between the buffy coat and the PMN fraction (2.96%). Pathway enrichment analysis of the DMPs identified biological pathways involved in the particular leukocyte lineage, consistent with perturbations during isolation. The comparisons between the buffy coat and the isolated fractions as a group using linear modeling yielded a small number of probes (∼29,000) with discordant methylation. Demethylation of probes in the buffy coat compared to derived cell lines was more common and was prevalent in shelf and open sea regions.

**Conclusion:** Buffy coat is representative of methylation patterns in WBC types in normal pregnancy. The differential methylations are consistent with perturbations during isolation of constituent cells and likely originate *in vitro* due to the physical stress during cell separation and are of no physiological relevance. These findings help the interpretation of DNA methylation profiling in pregnancy and numerous other conditions.

## Introduction

DNA methylation, the addition of a 5-methyl group to a cytosine nucleotide at a cytosine-phosphate-guanine (CpG) site, is an essential epigenetic modification implicated in switching on and off genes that control early mammalian embryogenesis, including development, differentiation, imprinting, and cellular function (Straussman et al., 2009; Portela and Esteller, 2010; Hodges et al., 2011; Jones, 2012). Many CpG sites are clustered in CpG islands which are flanked by shores and shelves (up to 2, and 2–4 kb from CpG islands, respectively) and are separated by “open sea” regions, which represent the rest of the genome. Tissue-specific DNA methylation tends to be observed within CpG shores rather than islands (Doi et al., 2009). DNA methylation frequently affects a gene promoter leading to gene silencing, while DNA methylation of the gene body indicates active transcription (Hellman and Chess, 2007).

Altered DNA methylation of regulatory regions has been shown to contribute to the control of proliferative, invasive, and immune tolerance mechanisms involved in oncogenesis (Klutstein et al., 2016; Kuss-Duerkop et al., 2018)— a disease process with many parallels to that of normal pregnancy—with the common goal of providing a nutrient supply and immune tolerance to a growing tumor and fetus, respectively. In our previous studies, we described a transient state of hypomethylation in maternal leukocyte DNA occurring in normal early pregnancy (White et al., 2012). We also have demonstrated that in preeclampsia—a pregnancy-specific hypertensive disorder clinically characterized by multisystem involvement and, commonly, proteinuria—maternal leukocyte DNA showed genome-wide differential methylation favoring hypermethylation compared with normotensive pregnant controls (White et al., 2013; White et al., 2016). Similar to other investigators, we have performed our studies on buffy coat, a mixed leukocyte cell population, which is obtained after centrifuging whole anticoagulated blood at low speeds (Houseman et al., 2015). The scientific rigor of such results critically depends on the ability to discern any experimentally introduced methylation changes.

The buffy coat of a healthy, non-pregnant individual contains white blood cells, leukocytes, which are comprised of 70% polymorphonuclear (PMN) leukocytes, also referred to as granulocytes (0.5–1% basophils, ∼65% neutrophils and 2–4% eosinophils), and roughly 30% mononuclear cells (3–8% monocytes, and 20–25% lymphocytes). Consequently, buffy coat comparisons may be confounded by shifts in cell type composition, which occur both in physiological conditions and during disease processes. This may be of particular importance for pregnancy, when an increase in the number of PMN leukocytes and monocytes, together with a decrease in the number of lymphocytes, dendritic cells, and natural killer (NK) cells occurs (Naccasha et al., 2001; Luppi et al., 2002a; Luppi et al., 2002b). Preeclampsia has been shown to be associated with an increase in the number of neutrophils (Järemo et al., 2000), along with activation of other PMN leukocytes and monocytes (Sacks et al., 1999). These shifts in white blood cell composition that occur both in normotensive and preeclamptic pregnancies could affect the DNA methylation results found in the buffy coat (Adalsteinsson et al., 2012). As methylation can vary at specific loci among individual cell types, the shifts in buffy coat cell composition, rather than shifts in cell-intrinsic methylation patterns, may cause methylation differences between buffy coat samples.

To date, limited work has addressed the stability and correlation of DNA methylation patterns in buffy coat compared with the different leukocyte fractions. Given our research focus on pregnancy and its complications, the objective of the current study was to assess to what extent the buffy coat methylome is representative of, or different from, the distinct cell types that it contains, namely PMN leukocytes and lymphocytes. To that end, we compared genome-wide methylation patterns in buffy coat to those of the PMN and lymphocytic fractions in the same pregnant individuals from blood samples collected within 24 h prior to delivery.

## Materials and Methods

### Sources for Cases and Blood Samples

Pregnant women were recruited prospectively at Mayo Clinic in Rochester, MN, United States A convenience sample of 30 ml of blood was drawn into an EDTA tube from normotensive pregnant women admitted for delivery and within the 24 h prior to giving birth. In each of 29 cases, the leukocytes were separated into three groups within 2 hours of collection. Using typical slow centrifugation, a buffy coat was made and immediately frozen. The remaining sample was further subdivided into a PMN and a lymphocytic fraction using a Ficoll gradient and subsequently frozen.

### Clinical Data

The medical records were abstracted for data including age, ethnicity, gravidity, body mass index (BMI), and gestational age (GA) at the time of delivery.

### DNA Extraction and Processing

In the buffy coat and PMN fraction, DNA was extracted using the AutoGenFlex DNA purification kit. In order to enrich the yield, manual extraction of DNA was performed for the lymphocytic fraction. Genomic DNA was quantified using a NanoDrop spectrophotometer, normalized with standard Pico Green methodology and plated. Bisulfite modification was performed using the EZ DNA Methylation Kit (Zymo Research).

### Methylation Assay

Plate maps were generated to determine the random location for each sample on the plate, as well as the samples that were run in duplicate. All samples were run in a single batch. Bisulfite-treated DNA was hybridized and imaged on an Illumina Infinium Human Methylation 450 K BeadChip that can detect methylation levels at 486,685 CpG dinucleotides across the genome and covers 96% of the CpG islands in the human genome.

### Quality Control

The samples were processed and then scanned using Illumina’s iScan instrumentation. The raw data were then analyzed using Illumina’s Genome Studio software (version 2011.1), with methylation module (version 1.9.0). Quality assessment of the array was conducted using the “Control Dashboard” in the Genome Studio software package, which includes a graphical inspection of the 10 types of embedded control probes: staining, extension, hybridization, target removal, bisulfite conversion, G/T mismatch, specificity, non-polymorphic controls, negative controls, and restoration controls.

Overall sample performance was determined by the total number of detected CpGs, the average detection *p* value across all CpG sites, and the distribution of average beta values for all CpGs. Call rates for each CpG site and sample were determined. Methylation sites and samples were excluded if the unreliable call rate (detection *p*-value) was greater than 5%. Technical replicate reproducibility was estimated by the Pearson correlation coefficient.

While all samples were bisulfite modified and run concurrently to avoid batch effects, multiple BeadChips were used, and may have variations in assay integrity leading to the “chip” effect. Thus, data were examined using principal components analysis and subsequent unsupervised hierarchical clustering of obtained components.

### Statistical Analysis

**FIGURE 1:**
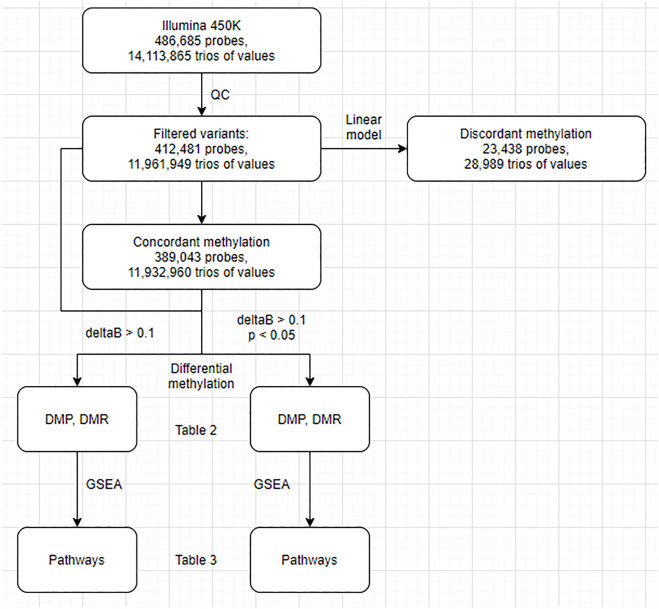
Flowchart of the analysis. DMP, differentially methylated probes; DMR, differentially methylated regions; GSEA, gene set enrichment analysis.

First, we conducted a standard differential methylation analysis to examine DNA methylation differences between the buffy coat and its PMN and lymphocytic fractions. Second, we aimed to differentiate between true differential methylation and discordant methylation, the latter being defined as methylation of specific CPGs in the buffy coat that did not match their methylation status in derived (PMN and lymphocytic) fractions (Figure 1). We used linear modeling to determine whether the linear combination of methylation profiles from PMN and lymphocytic fractions predicts the methylation profile of the buffy coat. Assuming that the methylation status of each CPG site in the buffy coat will agree with the methylation status in the derived fractions, we identified concordant (the presence of prediction/agreement) and discordant (the lack of prediction/agreement) CpGs. Third, we attempted to investigate the factors predicting discordance (e.g., presence of nearby SNPs, GC composition of probes, presence of motifs).

**FIGURE 2:**
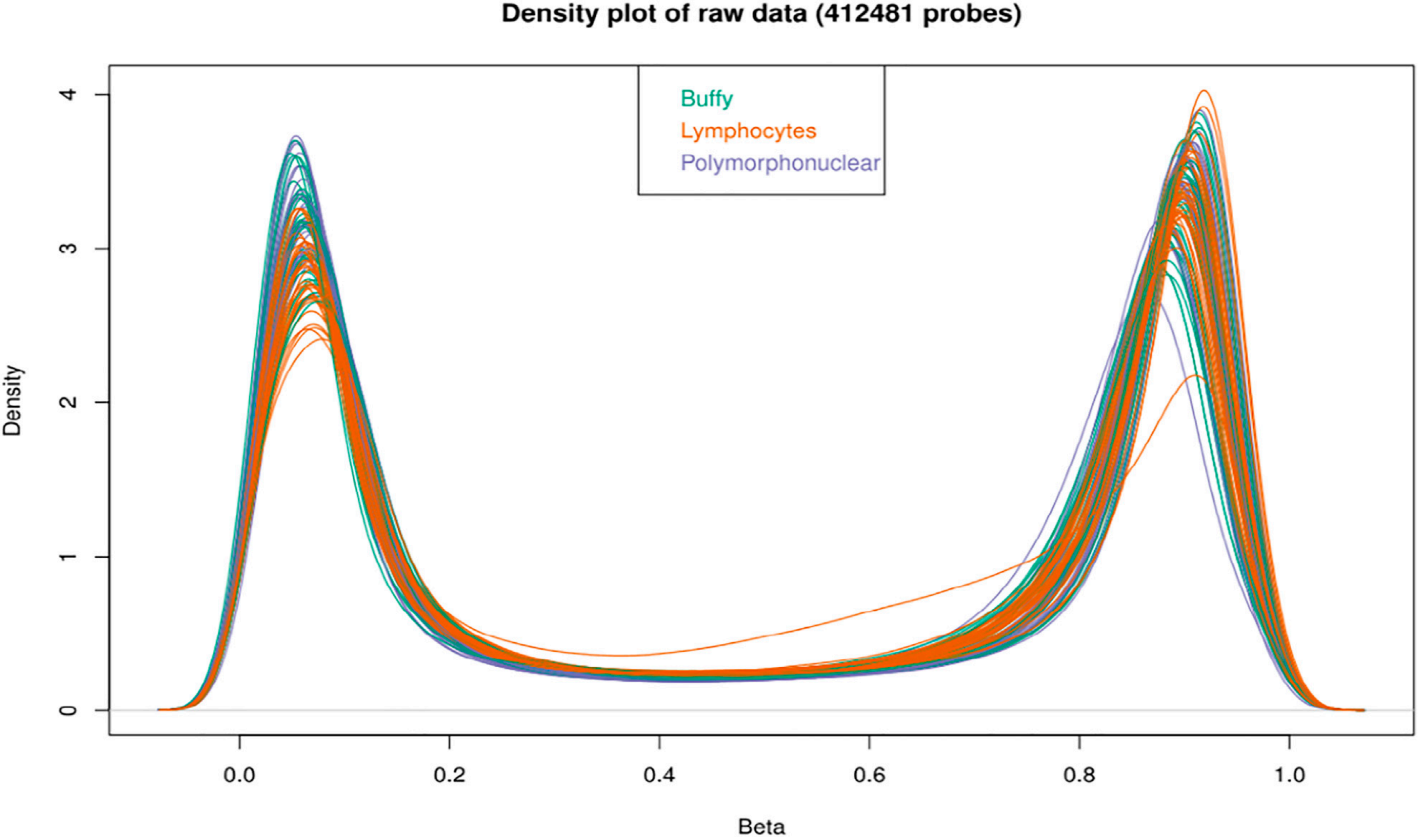
Density plot of samples raw beta distributions (412,481 probes); the buffy coat, polymorphonuclear leukocytes and lymphocytes showed a similar distribution of beta values. All the probes show an expected bimodal grouping at extreme ends.

Analysis of the differential methylation at the individual CpG probe level (DMP) was performed using the Limma package (version 3.30.13) (Smyth, 2004). To adjust for multiple comparisons, we used the Benjamini-Hochberg procedure of multiple testing, with  α=0.05 . Analysis of the differentially methylated regions (DMR) as aggregates of individual probes was performed using the DMRcate package (Peters et al., 2015). In DMRcate, a default false discovery rate (FDR) cutoff of 0.05 was used for CpG sites. The data generated by DMR and DMP were used as an input for Figure 2. Gene set enrichment analysis (GSEA) was performed using the Gometh method to avoid probes-per-gene bias (Young et al., 2010; Geeleher et al., 2013; Phipson et al., 2016). Another filtering criterion, in addition to statistical significance, was differences in beta (delta beta) larger than 0.1. For the purposes of enrichment and correlation analyses, we used the GRCh37 reference build. The mappability and uniqueness of probes was calculated using Bismap (Karimzadeh et al., 2018). Normalized beta values and sample annotations are available to the public at https://osf.io/324ak/?view_only = 5c1c7cf5b77a40d3bb29b7d9c418f763.

## Results

### Clinical Characteristics

The blood samples of 29 normotensive pregnancies were collected and analyzed. Patients were predominantly white, and the mean gestational age at delivery was 39.9 ± 1.4 weeks. Demographic and clinical characteristics are listed in Table 1.

**TABLE 1:** Study population demographics.

Demographics	All patients (*n* = 29)
Age, yrs (mean ± SD)	29.2 ± 6.0
White, *n* (%)	24 (82.8)
Gravida, *n* (%)	
1	13 (44.8)
>1	16 (55.2)
Parity, *n* (%)	
0	14 (48.3)
1	4 (13.8)
>1	11 (37.9)
BMI, (kg/m^2^), (mean ± SD)	29.3 ± 7.7
GA at delivery, weeks, (mean ± SD)	39.9 ± 1.4

BMI, body mass index; GA, gestational age; SD, standard deviation.

### Differential Methylation

**FIGURE 3:**
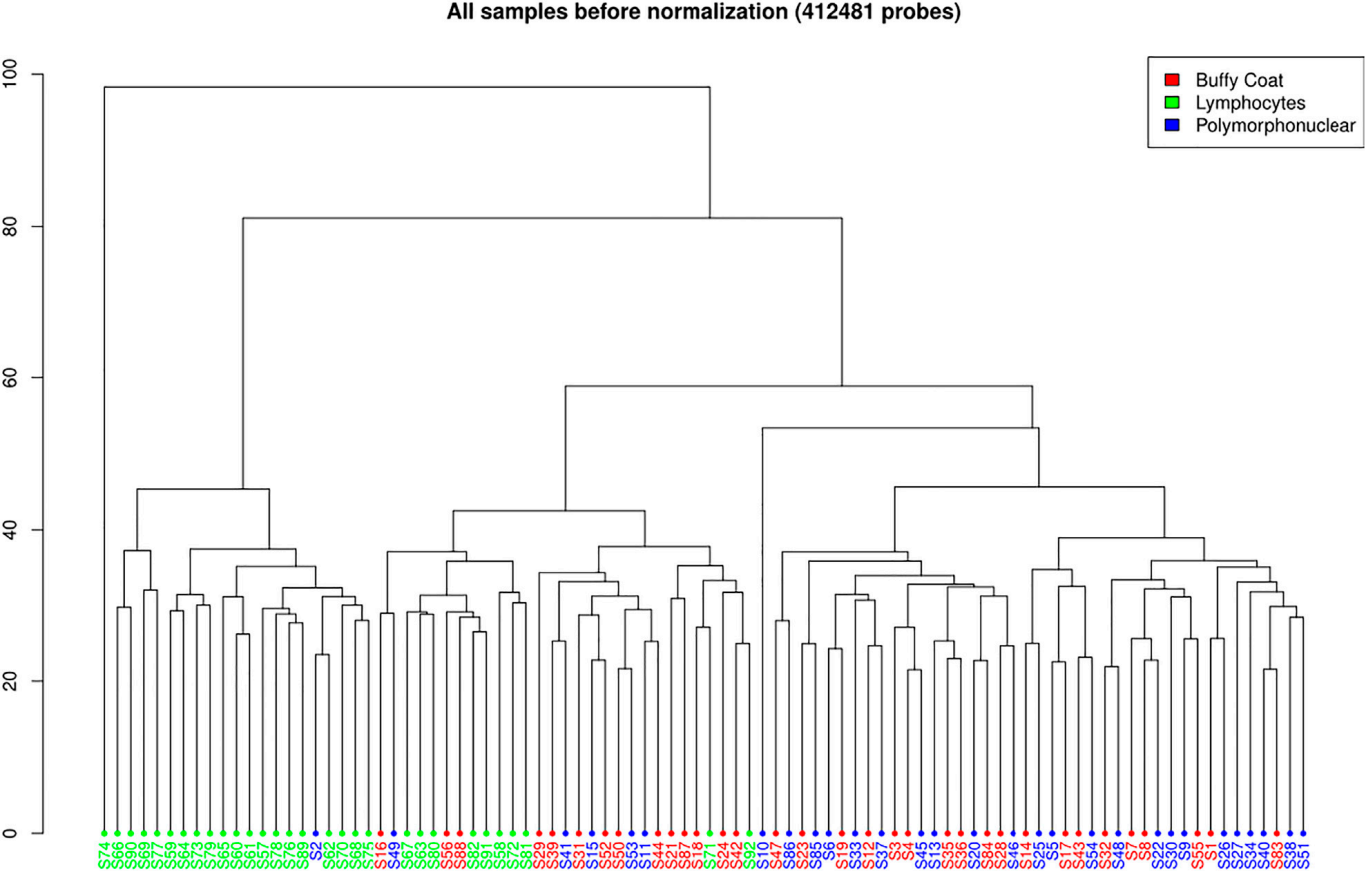
Hierarchal clustering of all samples before normalization (412,481 probes) on Illumina 450K data.

**FIGURE 4:**
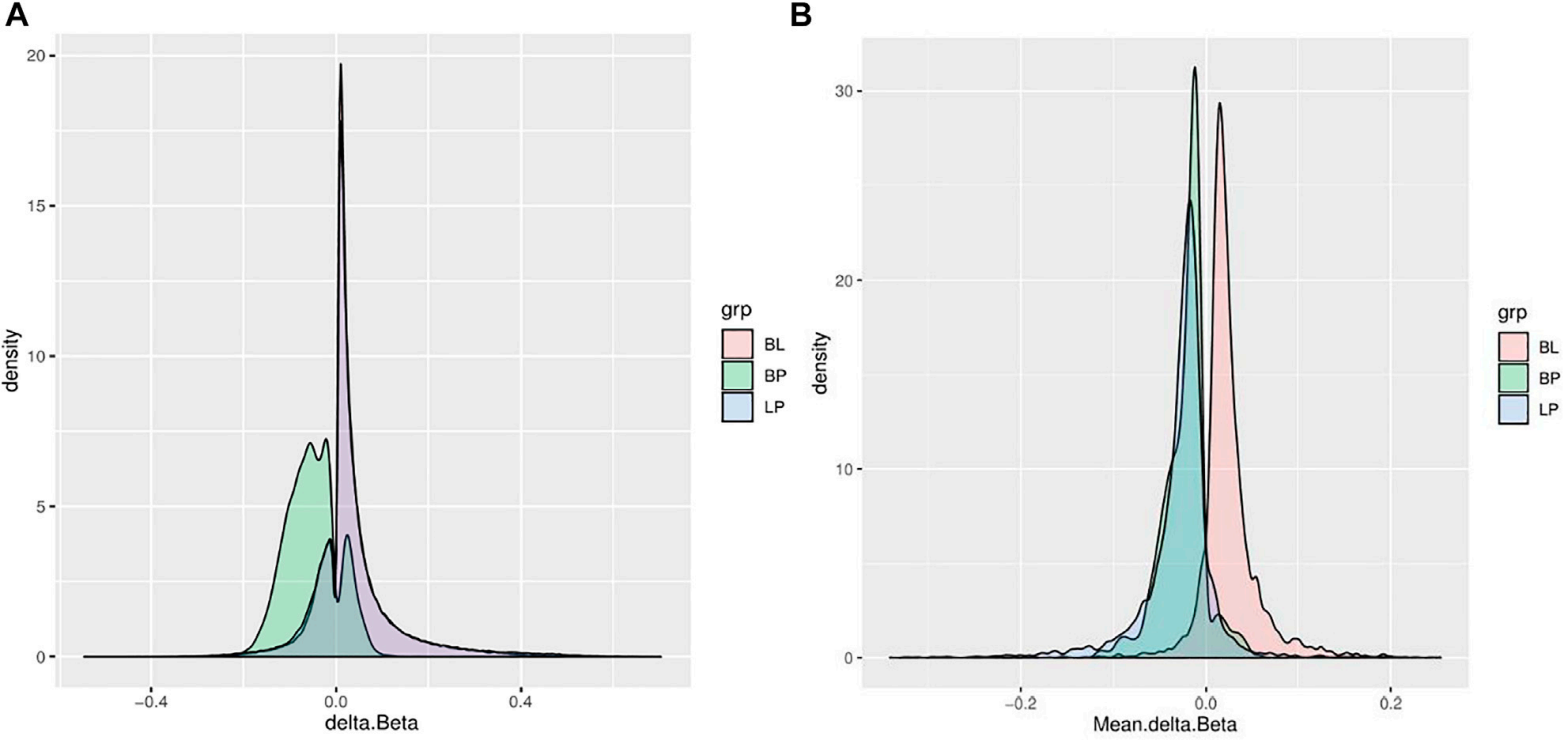
Probability density functions for the effect size between groups. These distributions are useful for selecting cutoffs for differential methylation. **(A)** Differences in beta values for differentially methylated probes. **(B)** Differences in mean delta beta for differentially methylated regions. BP, Comparison of buffy coat to polymorphonuclear leukocytes: BL, Comparison of buffy coat to lymphocytes; PL, Comparison of polymorphonuclear leukocytes to lymphocytes.

A total of 412,481 probes passed all the filters (Figure 3). A pairwise comparison between the buffy coat, PMN leukocytes, and lymphocytes was performed to identify differentially methylated probes (DMP). When considering the α=0.05  significance level with Benjamini-Hochberg multiple testing corrections, the greatest number of differentially methylated sites was found between PMN and lymphocytes (143,097; 34.69%), and the smallest between the buffy coat and PMN (12,207; 2.96%), as represented in Table 2. This finding is not unexpected, considering that the buffy coat contains predominantly PMN leukocytes, so in this case only a small proportion of other cells (mainly lymphocytes) was present and contributed to differential signals. The opposite rationale can be drawn for the comparison of buffy coat and lymphocyte fractions. Clustering in Figure 3 corroborates this with a clearly distinct lymphocyte fraction and an overlap of the other two fractions. The analysis was subsequently repeated for differentially methylated regions (DMR), but these results have moderate interpretability due to the variable sizes of a region, and they primarily served as an input for gene set enrichment analysis (GSEA). The magnitudes of differences for both the DMP and DMR analyses are shown in Figure 4A (delta beta) and 4 B (mean beta fold change). In addition, the number of differentially methylated probes/regions between the groups are shown when the applied threshold for delta beta was 0.1 (Original Analysis in Table 2).

**TABLE 2:** Number of differentially methylated probes and regions.

Differentially methylated	Significance filters	Original analysis			Without violating probe trios^a^		
		BP	BL	PL	BP	BL	PL
DMP	*p* < 0.05	12,207	125,962	143,097	13,926	124,251	143,048
	*p* < 0.05 and Δβ>0.1	2,450	20,271	26,334	2,963	20,303	26,495
DMR	*p* < 0.05	596	5,270	6,086	627	5,209	6,120
	*p* < 0.05 and Δβ>0.1	0	164	25	0	157	24
GSEA-DMP	*p* < 0.05	15	0	0	14	0	0
	*p* < 0.05 and Δβ>0.1	11	32	17	12	31	23
GSEA-DMR	*p* < 0.05	0	10	19	0	14	23
	*p* < 0.05 and Δβ>0.1	0	1	1	0	0	0

a Analysis of differentially methylated probes after elimination of outliers.

DMP, differentially methylated probes; DMR, differentially methylated regions; GSEA, gene set enrichment analysis.

BP, Comparison of buffy coat to polymorphonuclear leukocytes; BL, comparison of buffy coat to lymphocytes; PL, comparison of polymorphonuclear leukocytes to lymphocytes.

### Gene Set Enrichment Analysis

Gene set enrichment analysis **(**GSEA) was performed using either only *p* < 0.05, or by combining *p* < 0.05 with delta beta >0.1. When using only *p*-values, DMPs give enriched gene sets only for the buffy coat to polymorphonuclear leukocytes (BP) pairing, where the least differences in methylation are expected. Matching DMR analyses give opposite (and expected) results with no gene sets for the BP and a larger number of sets in the polymorphonuclear leukocytes to lymphocyte (PL) pairing. The pathways identified tended to be general in nature, e.g., *gene silencing by miRNA, mRNA binding involved in post-transcriptional gene silencing,* and *micro-ribonucleoprotein complex* (Table 2). In contrast, when we used delta beta values as an additional filter, GSEA generated several pathways for DMP and virtually no pathways for DMR, and the resulting pathways were indicative of the leukocyte lineages, e.g., *innate immune response, inflammatory response,* and *neutrophil degranulation* (Table 3).

**TABLE 3:** Gene sets obtained.

Method	Sig. Filters	Original analysis			Without violating probe trios*		
		BP	BL	PL	BP	BL	PL
GSEA-DMP	*p* < 0.05	1.neutrophil degranulation					
		2.specific granule membrane			1.neutrophil degranulation		
		3.specific granule lumen			2.specific granule membrane		
		4.signal transduction			3.tertiary granule membrane		
		5.tertiary granule membrane			4.external side of plasma membrane		
		6.external side of plasma membrane			5.actin binding		
		7.inflammatory response			6.immune response		
		8.cellular response to lipopolysaccharide			7.signal transduction		
		9.humoral immune response			8.specific granule lumen		
		10.innate immune response			9.innate immune response		
		11.positive regulation of interleukin-10 production			10.positive regulation of interleukin-10 production		
		12.actin binding			11.humoral immune response		
		13.plasma membrane			12.tertiary granule lumen		
		14.antimicrobial humoral response			13.positive regulation of I-kappaB kinase/NF-kappaB signaling		
		15.tertiary granule lumen			14.cellular response to lipopolysaccharide		
	*p* < 0.05 & Δβ>0.1	1.neutrophil degranulation	1.neutrophil degranulation	1.neutrophil degranulation	1.neutrophil degranulation	1.neutrophil degranulation	1.neutrophil degranulation
		2.specific granule lumen	2.inflammatory response	2.inflammatory response	2.specific granule lumen	2.inflammatory response	2.inflammatory response
		3.specific granule membrane	3.specific granule membrane	3.specific granule membrane	3.specific granule membrane	3.specific granule membrane	3.innate immune response
		4.antimicrobial humoral response	4.innate immune response	4.innate immune response	4.cytosol	4.innate immune response	4.specific granule membrane
		5.innate immune response	5.cytokine-mediated signaling pathway	5.signal transduction	5.antimicrobial humoral response	5.cytokine-mediated signaling pathway	5.signal transduction
		6.tertiary granule lumen	6.immune response	6.cytokine-mediated signaling pathway	6.tertiary granule lumen	6.signal transduction	6.cytokine-mediated signaling pathway
		7.cytosol	7.signal transduction	7.immune response	7.innate immune response	7.immune response	7.immune response
		8.tertiary granule membrane	8.specific granule lumen	8.lipopolysaccharide-mediated signaling pathway	8.defense response to bacterium	8.specific granule lumen	8.lipopolysaccharide-mediated signaling pathway
		9.defense response to bacterium	9.tertiary granule membrane	9.tertiary granule membrane	9.protein binding	9.lipopolysaccharide-mediated signaling pathway	9.tertiary granule membrane
		10.azurophil granule lumen	10.lipopolysaccharide-mediated signaling pathway	10.external side of plasma membrane	10.azurophil granule lumen	10.tertiary granule membrane	10.cellular response to lipopolysaccharide
		11.protein binding	11.positive regulation of GTPase activity	11.chemotaxis	11.tertiary granule membrane	11.positive regulation of GTPase activity	11.external side of plasma membrane
			12.B cell receptor signaling pathway	12.signaling receptor activity	12.endoribonuclease activity	12.positive regulation of I-kappaB kinase/NF-kappaB signaling	12.positive regulation of I-kappaB kinase/NF-kappaB signaling
			13.cellular response to lipopolysaccharide	13.cellular response to lipopolysaccharide		13.cellular response to lipopolysaccharide	13.B cell receptor signaling pathway
			14.positive regulation of I-kappaB kinase/NF-kappaB signaling	14.positive regulation of GTPase activity		14.B cell receptor signaling pathway	14.positive regulation of interleukin-2 biosynthetic process
			15.secretory granule membrane	15.positive regulation of I-kappaB kinase/NF-kappaB signaling		15.secretory granule membrane	15.cell surface
			16.cell surface	16.secretory granule membrane		16.T cell activation	16.signaling receptor activity
			17.T cell activation	17.T cell activation		17.chemotaxis	17.chemotaxis
			18.perinuclear region of cytoplasm			18.cell surface	18.secretory granule membrane
			19.chemotaxis			19.negative regulation of tumor necrosis factor production	19.cell-cell adhesion
			20.Golgi apparatus			20.tertiary granule lumen	20.T cell activation
			21.negative regulation of tumor necrosis factor production			21.cellular defense response	21.positive regulation of GTPase activity
			22.tertiary granule lumen			22.T cell costimulation	22.focal adhesion
			23.cellular defense response			23.adaptive immune response	23.negative regulation of tumor necrosis factor production
			24.T cell costimulation			24.cytosol	
			25.adaptive immune response			25.platelet activation	
			26.platelet activation			26.GTPase activator activity	
			27.regulation of immune response			27.Golgi apparatus	
			28.GTPase activator activity			28.regulation of immune response	
			29.cytosol			29.perinuclear region of cytoplasm	
			30.external side of plasma membrane			30.extracellular exosome	
			31.Golgi membrane			31.Golgi membrane	
			32.extracellular exosome				
GSEA-DMR	*p* < 0.05		1.mRNA binding involved in posttranscriptional gene silencing	1.gene silencing by miRNA		1.mRNA binding involved in posttranscriptional gene silencing	1.gene silencing by miRNA
			2.micro-ribonucleoprotein complex	2.mRNA binding involved in posttranscriptional gene silencing		2.micro-ribonucleoprotein complex	2.mRNA binding involved in posttranscriptional gene silencing
			3.gene silencing by miRNA	3.micro-ribonucleoprotein complex		3.gene silencing by miRNA	3.micro-ribonucleoprotein complex
			4.extracellular space	4.extracellular space		4.extracellular space	4.extracellular space
			5.DNA-binding transcription activator activity, RNA polymerase II-specific	5.DNA-binding transcription activator activity, RNA polymerase II-specific		5.DNA-binding transcription activator activity, RNA polymerase II-specific	5.extracellular region
			6.uterus development	6.extracellular region		6.extracellular region	6.DNA-binding transcription activator activity, RNA polymerase II-specific
			7.DNA-binding transcription factor activity, RNA polymerase II-specific	7.integral component of plasma membrane		7.uterus development	7.integral component of plasma membrane
			8.extracellular region	8.collagen-containing extracellular matrix		8.embryonic forelimb morphogenesis	8.collagen-containing extracellular matrix
			9.odontogenesis	9.DNA-binding transcription factor activity, RNA polymerase II-specific		9.odontogenesis	9.sequence-specific DNA binding
			10.embryonic forelimb morphogenesis	10.embryonic forelimb morphogenesis		10.positive regulation of neuron differentiation	10.DNA-binding transcription factor activity, RNA polymerase II-specific
				11.dopaminergic neuron differentiation		11.collagen-containing extracellular matrix	11.embryonic forelimb morphogenesis
				12.homophilic cell adhesion via plasma membrane adhesion molecules		12.dopaminergic neuron differentiation	12.homophilic cell adhesion via plasma membrane adhesion molecules
				13.sequence-specific DNA binding		13.integral component of plasma membrane	13.dopaminergic neuron differentiation
				14.uterus development		14.DNA-binding transcription factor activity, RNA polymerase II-specific	14.uterus development
				15.negative regulation of sprouting angiogenesis			15.negative regulation of sprouting angiogenesis
				16.calcium ion binding			16.chemical synaptic transmission
				17.extracellular matrix organization			17.cell junction
				18.neuropeptide signaling pathway			18.neuropeptide signaling pathway
				19.cell junction			19.extracellular matrix organization
							20.pancreas development
							21.calcium ion binding
							22.DNA-binding transcription factor activity
							23.growth factor activity
	*p* < 0.05 & Δβ>0.1		1.neutrophil degranulation	1.neutrophil degranulation			

### Consistency of Results Between Leukocyte Fractions and the Buffy Coat

Under the assumption that the buffy coat is mostly the weighted sum of PMN and lymphocytes, buffy coat methylations are essentially estimated twice. We took advantage of the redundant methylation information to test whether we could extract the ratio of PMN and lymphocytes in the peripheral blood. Without additional constraints, we fit the model *buffy coat ∼ lymphocytes + PMN leukocytes + error* for each subject and obtained coefficients for both lymphocytes and PMN summing to 0.98–0.99. This shows that the model is valid and reflects the physiological setup. The coefficients were interpreted as the differential blood count, with an expectancy of 20–40% lymphocytes and 40–80% PMN leukocytes.

**FIGURE 5:**
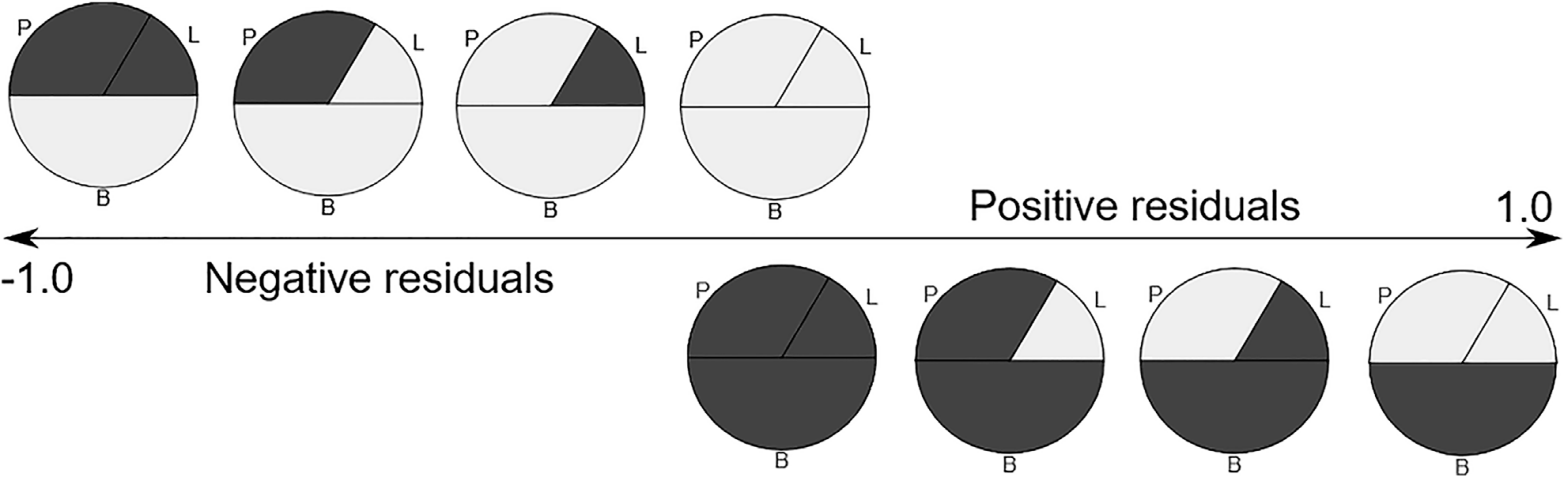
The expected residuals of the linear model based on the methylation state of each fraction. Methylated regions are shown in dark gray. P-polymorphonuclears, L-lymphocytes, B-buffy coat.

**FIGURE 6:**
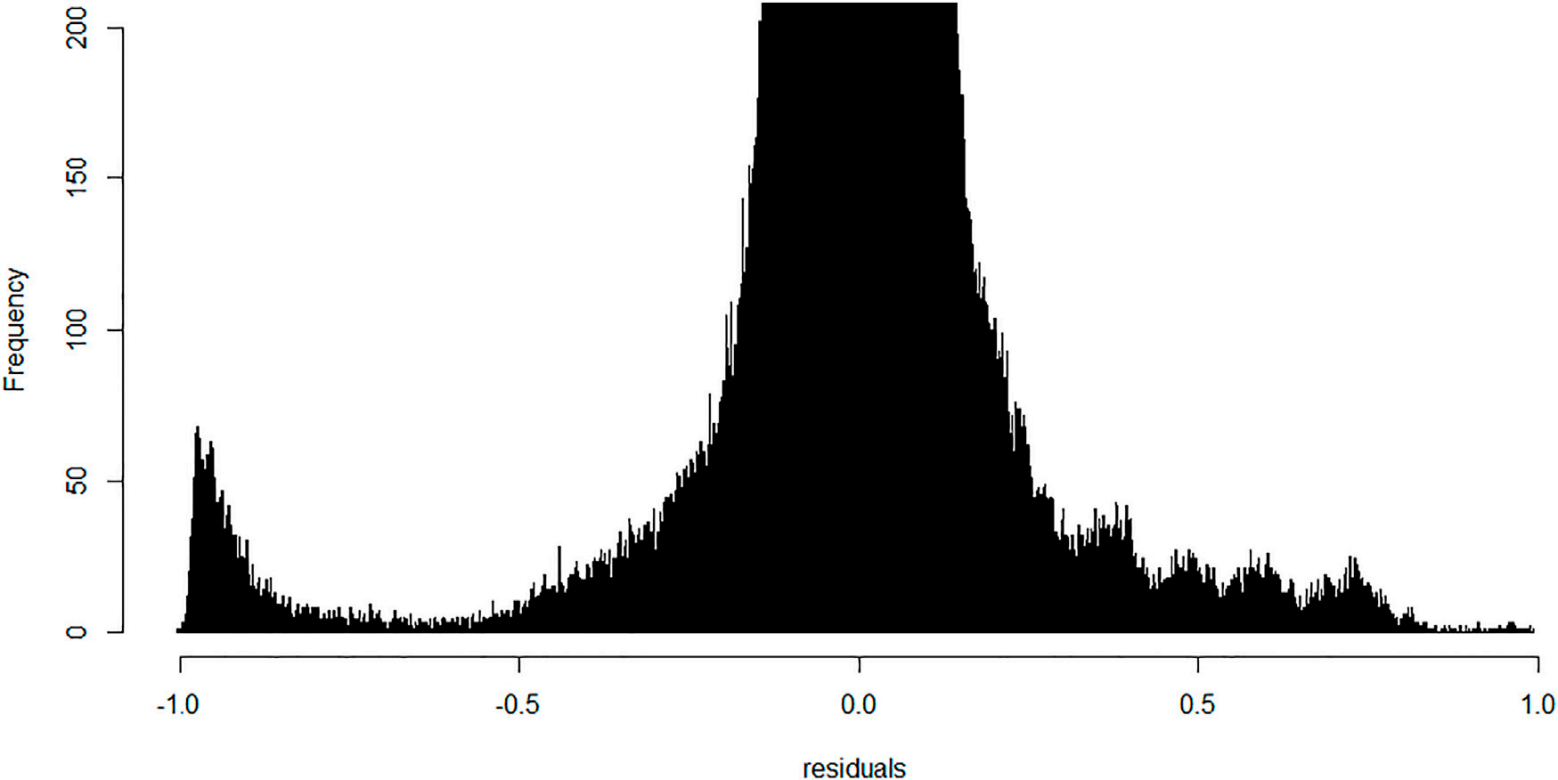
Frequency of probes generated residuals after application of the fitted linear model; bin width is 0.001. Y-range is limited from 0–200 to allow visualization of important minority groups. Most of the probes centered around zero with a small peak in the furthest negative region indicative of a cluster of probes methylated in polymorphonuclears and lymphocytes but not in the original buffy coat.

We next performed linear modeling to determine whether the weighted combination of methylation profiles from isolated cellular fractions predicts the methylation profile of the buffy coat and identified the discordant CpG probes where the weighted combination deviates from methylation measured on the buffy coat. We applied the fitted model to each probe and each trio of samples and calculated the residuals (Figure 5). Although most probes centered around zero in a unimodal distribution, there was a prominent peak around the negative extreme (Figure 6), which corresponds to CpGs that are less methylated in the buffy coat than predicted from the relatively high CpG methylation in constituent fractions. Positive residuals correspond to methylated loci in buffy coat for which either of the fractions is hypomethylated and we can see several clusters between 0.3 and 0.8. Notably, the negative peak at the far left is dominant and has no matching positive counterpart. These discordant values with absolute residuals larger than expected could be a consequence of random probe errors or may be a result of physical/chemical stress occurring during cell separation, thus leading to methylation in isolated cellular fractions of particular site(s), which were not methylated in the initial, pre-separation analysis of the buffy coat.

For subsequent analysis, we calculated the *p* < 0.05 cutoff using Bonferroni correction (z-score 5.29) and labeled as discordant all probes that fell outside of this range as outliers when standardized. The total number of discordant probe trios was 28,989 (0.24%) out of n*412,481 (*n* = 29) included in the model. These values were filtered out of the data from all three types of samples. All analyses were then repeated with the new “filtered” dataset (called without discordant probes) to determine whether the differentially methylated regions or probes were actually false positives. We found that, regardless of the filtering criteria used, the changes were minor in terms of DMP and DMR (Table 2 left, without discordant probes) and did not seem to affect the top scoring GSEA sites (Table 2 right).

### Analysis of Discordant Probes

The number of discordant probes for which prediction of the methylation in the buffy coat was discordant from the prediction based on constituent fractions (PMN leukocytes and lymphocytes) was 23,438 (4.83% of probes). The remaining 389,043 probes that passed filters were labeled as “concordant.” We hypothesized that there would be something in the design or location of those probes that would predict discordance. We downloaded the Illumina 450K manifest and analyzed the properties that differed between the two datasets.

After intersecting the locations of discordant probes with the unique Bismap ranges, only 11 of 23,438 discordant probes proved to map to non-unique segments in the bisulfite-converted reference, which are expected to yield more reliable results. On the other hand, 212 of 389,043 concordant probes proved to be multi-mapped in the concordant probes. Both types of probes had identical uniqueness (99.95%) rounded to four significant digits, suggesting that systematic mis-mapping does not explain discordance. Interestingly, 11,730 of 73,031 (16.06%) probes that were filtered out by ChAMP default QC mapped to non-unique 50-mers according to this methodology. This high uniqueness of virtually all the probes that passed filters indicates that the probes are hybridizing with the expected sequences and that the discordance is an *in-vitro*, pre-measurement phenomenon.

We also noted a prominent difference in guanine-cytosine (GC) content, and the discordant probes in the linear model had approximately 3% lower GC. One of the probes was discordant in 14 of 29 trio samples but had only a moderate GC content of 55%. Similarly, many of the probes with a higher error rate had lower GC values.

**FIGURE 7:**
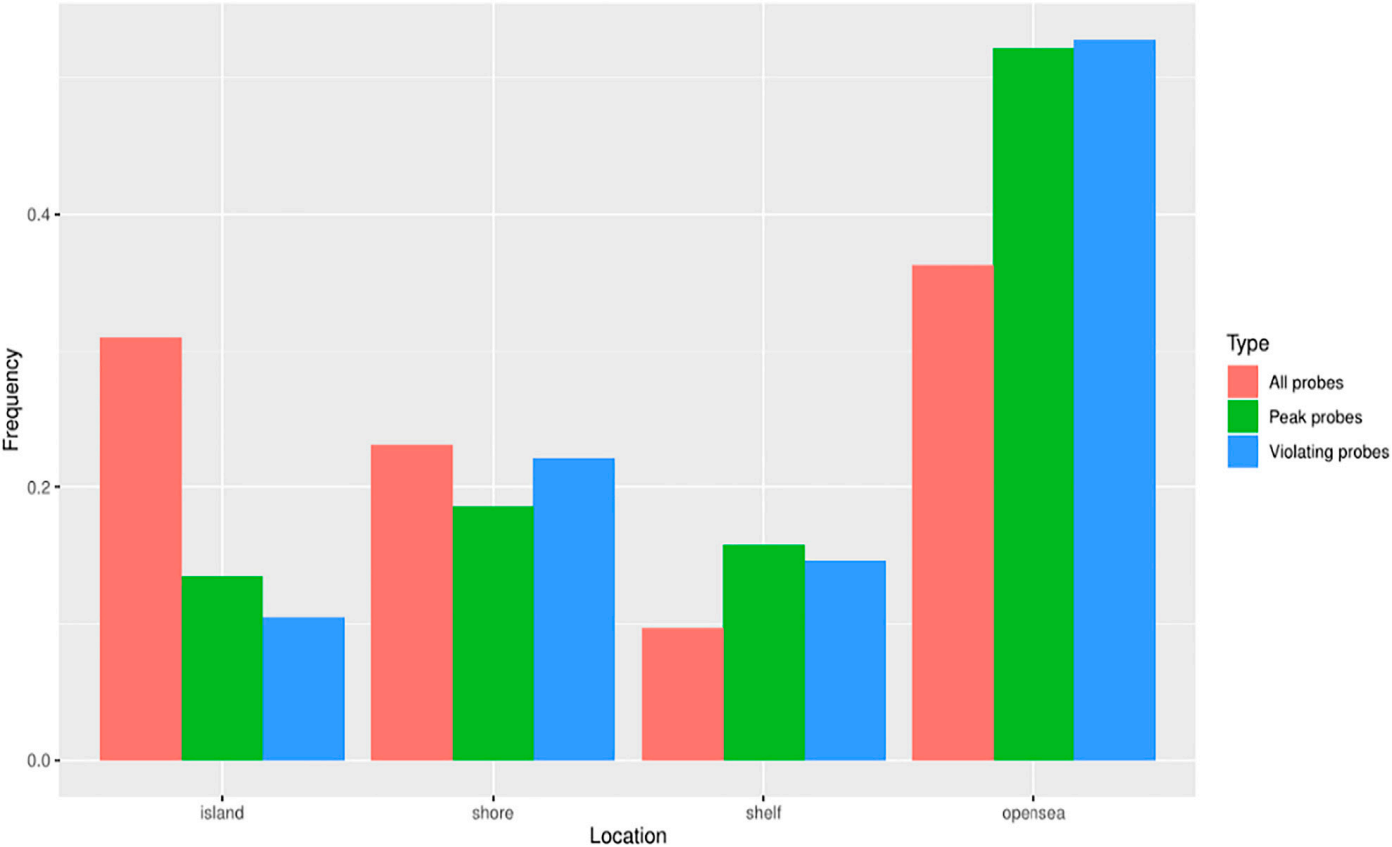
Genomic loci classification of the probes. Violating and peak probes were less frequently located in CpG islands compared to all probes. Violating probes - probes with at least one discordant beta value. Peak probes - probes appearing in the peak of residuals below −0.6.

As mappability and GC content strongly differ along the genome, and presumably correlate with the presence of CpG islands, we investigated the location of discordant probes and tried to determine any preference for genomic loci relative to CpG islands. To avoid effect of overrepresentation of individual probes without the understanding of the underlying process, we counted each probe only once regardless of the number of occurrences in the investigated groups. Resorting to the standard four groups—CpG islands, shores, shelves, and open sea—we plotted and tested the proportion of groups for all probes (412,481), discordant probes (23,438), and the subset of probes giving the peak of residuals below −0.6 (peak probes, 4,535). We noted that the genomic location of both discordant groups (discordant and peak probes) was significantly different from the group of all probes (Figure 7; Table 4). These differences indicated that the discordant trios were most likely to be located in open sea regions, and least likely to be present in CpG islands.

**TABLE 4:** *p*-values obtained by chi-squared proportion tests on different types of discordant probes with the pool of all probes, stratified by the CpG region. All results are significant at α = 0.05

Comparison with all probes	Islands	Shore	Shelf	Open sea
Violating probes	< 2.2e-16	9.479e-08	< 2.2e-16	< 2.2e-16
Peak probes	< 2.2e-16	1.726e-15	< 2.2e-16	< 2.2e-16

## Discussion

Our present study reports several novel findings obtained through a comparative analysis of the methylation profile in buffy coat versus PMN and lymphocyte cell lines. First, the buffy coat methylation profile was representative of methylation patterns of derived cell lines. We characterized the genome-wide methylation profile of the buffy coat, PMN and the lymphocytic fractions drawn from the same individual in 29 normotensive pregnancies across >450,000 CpG sites in genes across the entire genome. We performed pairwise comparisons that yielded a number of probes that are differentially methylated, but with relatively small differences in beta values. We found a very small percentage of differentially methylated CpG sites when the buffy coat was compared to the PMN fraction (2.96%) and a greater percentage of differentially methylated CpG sites between PMN and lymphocytes (34.69%), consistent with the fact that the PMN fraction is the main constituent of the buffy coat Second, differential methylation occurred in biological pathways that are specific for the derived cell lines, such as neutrophil degranulation and cytokine-mediated signaling pathways, consistent with the sensitivity of these cell-type specific pathways to perturbations during the cell separation process. Third, using a linear model we were able to identify approximately 29,000 probes for which the prediction of CpG methylation within the buffy coat is discordant from the prediction from constituent fractions. These discordant probes had a lower GC content, and the CpG sites involved also preferentially affected the open sea and shelf regions; discordant methylation seems to be an *in vitro* phenomenon and likely due to the separation process of the buffy coat.

To date, few studies have provided comparative analyses of different epigenetic profiles across different blood fractions in pregnancy. One notable example is a study of the cord blood, which showed that methylation in whole blood is reasonably comparable to buffy coat in a small number (*n* = 8) of paired samples (Dou et al., 2018). However, the concordance between special cell populations (such as lymphocytes or monocytes) with either whole blood or buffy coat was not studied. To address cell type composition of complex tissues, we have previously developed and characterized *in silico* epigenomic deconvolution methods (Onuchic et al., 2016; Decamps et al., 2020) that infer DNA methylation of constituent cell types by minimizing residuals.

We now extend residual analysis to address the complexity of the blood samples with their multiple cell types by providing a novel approach to analyzing and interpreting methylation profiles from the tissue of origin (whole blood, buffy coat) and derived cell lines (PMN and lymphocyte cell lines). Negative residuals in the overall linear model indicate that at least one of the fractions changed its state from unmethylated to methylated in derived cell lines (i.e., PMN and lymphocytes), while the positive residuals representing the opposite process, were few. Most residuals in the interval between −0.7 and 0.7 seem balanced, in accordance with the expected normal distribution as a result of unobserved variation. On the other hand, close to 50% of discordant probes are grouped in a clear peak below −0.8 and have no substantial positive counterpart, i.e., the same probe being methylated in the buffy coat, but unmethylated in derived cell line(s), indicating strong preference towards methylation in PMN and lymphocytes. This targeted DNA inactivation, preferentially targeting isolated CpGs in the open-sea regions, could be the result of the separation process, and the mechanical/chemical stress exerted on the cells removed from their normal medium.

While our study was performed on a relatively small sample size, a pairwise comparison of samples drawn from the same individual should limit the effects of potential confounders. We studied only normotensive pregnancies within 24 h prior to delivery, thus limiting the extrapolation of our results to other conditions with vastly different white blood cell composition or with selective methylation of a certain white blood cell fraction.

Our results have the potential to improve rigor and reproducibility of studies involving epigenomic profiling of buffy coat samples. For example, in the context of one of our previous studies, we identified a state of transient hypomethylation in normal early pregnancy compared with non-pregnancy (White et al., 2012). This hypomethylation would spontaneously revert after delivery. Our sample included patients close to delivery, suggesting a potential tendency towards global hypomethylation. DNA methylation can be affected by many factors including age (Bell et al., 2012), race, BMI, smoking, gestational diabetes (Wu et al., 2018), and preeclampsia (White et al., 2013). The patients in our study had healthy normotensive pregnancies, with a similar age range, a relatively homogeneous ethnicity, and free of major medical comorbidities that could affect DNA methylation. Some fetal DNA contamination was possible, but it likely represented a very small fraction (up to 6% of total measured DNA) (Bischoff, 2002) and, therefore, it was unlikely to have significantly changed our results. However, in our original study, we could not firmly establish the relation between the DNA methylation profiles in buffy coat samples and that of constituent cell types. Our current results help establish this relation, thus improving both the rigor and biological interpretability of our results. Moreover, our results will help integrate results obtained from buffy coat samples and those obtained from profiling of isolated constituent cell types. Finally, our results are not limited to studies of pregnancy and have implications for numerous other studies involving DNA methylation profiling of buffy coat samples and constituent cell types.

A potential limitation of this study is that we did not account for the effect of DNA extraction techniques. DNA was isolated in samples using the AutoGenFlex DNA purification kit for the buffy coat and the neutrophil fraction, and manual extraction of DNA was performed for the lymphocytic fraction. The use of different methods for DNA isolation may affect methylation results, although the methylation mark is considered quite stable, but this is currently minimally discussed in the literature (Hjorthaug et al., 2018). Blood sample processing techniques, such as using Ficoll density centrifugation, can also potentially confound methylation. Finally, we used the Illumina 450K array, which covers only ∼2% of total CpGs within the human genome. The Illumina Infinium Human Methylation 450, however, controls for the confounding and takes SNPs into account when analyzing output data. Future directions include using a different sample cohort and methodology to confirm our results. We hope to reproduce our findings in a larger, independent sample, and in a different study population that would confirm the stability of the methylome in the buffy coat and its sub-fractions.

Despite these limitations, our results demonstrate that the buffy coat methylation profile is representative of the methylation patterns in white blood cell types in normal pregnancy obtained using Illumina Human Methylation 450 BeadChip. Small differences in the buffy coat composition may confound the methylation analysis at a very small number of CpG sites, but this is not likely to affect most results. Current methods to adjust for cellular heterogeneity, either by excluding these differentially methylated genes, or, better yet, adjusting methylation data to account for these differences in buffy coat composition, improve the robustness of methylome analysis in buffy coat. Overall, our results support DNA methylation profiling of buffy coats as an acceptable approach for epigenomic profiling in pregnancy research and suggest that separation is likely only needed when studying lineage-specific diseases.

## Data Availability

The datasets presented in this study can be found in an online repository. The name of the repository and accession link can be found below: OSF, https://osf.io/324ak/?view_only=5c1c7cf5b77a40d3bb29b7d9c418f763.
